# Multimodal artificial intelligence-based pathogenomics improves survival prediction in oral squamous cell carcinoma

**DOI:** 10.1038/s41598-024-56172-5

**Published:** 2024-03-07

**Authors:** Andreas Vollmer, Stefan Hartmann, Michael Vollmer, Veronika Shavlokhova, Roman C. Brands, Alexander Kübler, Jakob Wollborn, Frank Hassel, Sebastien Couillard-Despres, Gernot Lang, Babak Saravi

**Affiliations:** 1 Department of Oral and Maxillofacial Plastic Surgery, University Hospital of Würzburg, 97070 Würzburg, Franconia Germany; 2 Department of Oral and Maxillofacial Surgery, Tuebingen University Hospital, Osianderstrasse 2-8, 72076 Tuebingen, Germany; 3 Maxillofacial Surgery University Hospital Ruppin-Brandenburg, Fehrbelliner Straße 38, 16816 Neuruppin, Germany; 4 Department of Anesthesiology, Perioperative and Pain Medicine, Brigham and Women’s Hospital, Harvard Medical School, Boston, USA; 5 Department of Spine Surgery, Loretto Hospital, Freiburg, Germany; 6 Institute of Experimental Neuroregeneration, Paracelsus Medical University, 5020 Salzburg, Austria; 7 Austrian Cluster for Tissue Regeneration, Vienna, Austria; 8 Department of Orthopedics and Trauma Surgery, Medical Center - University of Freiburg, Faculty of Medicine, University of Freiburg, Freiburg, Germany

**Keywords:** Oral cancer, Oral squamous cell carcinoma, Survival, Artificial intelligence, Deep learning, Machine learning, Pathogenomics, Multimodal prediction, Cancer, Diseases, Health care, Medical research, Oncology

## Abstract

In this study, we aimed to develop a novel prognostic algorithm for oral squamous cell carcinoma (OSCC) using a combination of pathogenomics and AI-based techniques. We collected comprehensive clinical, genomic, and pathology data from a cohort of OSCC patients in the TCGA dataset and used machine learning and deep learning algorithms to identify relevant features that are predictive of survival outcomes. Our analyses included 406 OSCC patients. Initial analyses involved gene expression analyses, principal component analyses, gene enrichment analyses, and feature importance analyses. These insights were foundational for subsequent model development. Furthermore, we applied five machine learning/deep learning algorithms (Random Survival Forest, Gradient Boosting Survival Analysis, Cox PH, Fast Survival SVM, and DeepSurv) for survival prediction. Our initial analyses revealed relevant gene expression variations and biological pathways, laying the groundwork for robust feature selection in model building. The results showed that the multimodal model outperformed the unimodal models across all methods, with c-index values of 0.722 for RSF, 0.633 for GBSA, 0.625 for FastSVM, 0.633 for CoxPH, and 0.515 for DeepSurv. When considering only important features, the multimodal model continued to outperform the unimodal models, with c-index values of 0.834 for RSF, 0.747 for GBSA, 0.718 for FastSVM, 0.742 for CoxPH, and 0.635 for DeepSurv. Our results demonstrate the potential of pathogenomics and AI-based techniques in improving the accuracy of prognostic prediction in OSCC, which may ultimately aid in the development of personalized treatment strategies for patients with this devastating disease.

## Introduction

Oral cancer is a significant global health concern with high morbidity and mortality rates. Oral squamous cell carcinoma (OSCC), the most common type of oral cancer, results in an estimated 378,000 new diagnoses and over 177,000 deaths worldwide annually^1^. OSCC is commonly associated with unhealthy habits such as alcohol abuse, tobacco use, and chewing betel nuts, as well as human papillomavirus (HPV) infection^2^. The development of OSCC is generally asymptomatic in the early stages, leading to late diagnosis, extensive lesions, and potential metastases^3^. Despite intervention with advanced treatment regimens, the survival rate of OSCC has not significantly improved in recent decades, underlining the limitations of current prognostic methods^4^. These traditional approaches, primarily based on clinicopathological factors such as demographic variables, tumor size, lymph node involvement, and metastasis, often fail to capture the complex biological heterogeneity of OSCC, leading to suboptimal treatment stratification and prognostication^5^.

Prognostic markers are urgently required to better adjust treatment intensity and avoid serious complications caused by overtreatment. The current gold standard for cancer diagnosis involves the manual examination of H&E-stained slides by pathologists^6^. However, recent advances in deep learning for digital pathology have allowed for the use of whole-slide images (WSIs) for computational image analysis tasks, such as cellular segmentation and tissue classification^7^. Genomic data can provide a deeper molecular characterization of the tumor, offering the potential for prognostic and predictive biomarker discovery.

The utilization of unimodal input, which involves relying on data from a single resource, fails to fully exploit the potential benefits of incorporating more extensive information from other aspects of patients that may impact their overall survival time^8^. Current survival prediction in oncology often relies on traditional methods like the Kaplan–Meier estimator or Cox proportional hazards (Cox-PH) model. While these approaches have been the cornerstone of cancer prognosis, they primarily depend on limited variables such as patient demographics, tumor stage, and histopathological risk factors. This conventional methodology lacks the capability to account for the vast heterogeneity and complex biological mechanisms underlying different cancer types, including OSCC^9^. Recent research findings suggest that leveraging multi-omics data of cancer can significantly enhance the accuracy of non-small-cell lung cancer subtype classification compared to using a single modality approach^10^. Multimodal survival prediction is a sophisticated method used for biomarker discovery, patient stratification, and therapeutic response prediction^9^. Artificial intelligence-processed pathogenomics is a relatively new research field that combines genomics and pathology and has shown promise in identifying novel biomarkers and therapeutic targets for cancer^11^. Recent studies have demonstrated the potential of pathogenomics in predicting the survival of patients with different cancer types^11^. The increasing availability of high-throughput, multidimensional data from initiatives like the cancer genome atlas (TCGA) has revolutionized the field of cancer research. However, the complexity and volume of such data exceed the capabilities of traditional survival analysis methods. This gap has paved the way for the integration of advanced AI techniques, especially deep learning (DL), in analyzing complex clinical and genomic data. Models combining neural network architectures with the Cox-PH model, such as DeepSurv and Cox-nnet, have shown promise in outperforming traditional models by leveraging more complex, non-linear relationships in the data^12^. These developments underscore the potential of a multimodal approach, integrating clinical characteristics with diverse omics data, for enhancing cancer prognosis predictions. Particularly for OSCC, where traditional prognostic models have limitations, leveraging artificial intelligence (AI)-processed pathogenomics—an innovative field that combines genomics and pathology—holds great promise. This approach, relatively unexplored in OSCC, has shown potential in other cancer types for improving survival prediction accuracy^9,12^. Thus, utilizing a multimodal data integration strategy, which includes clinical data, histology, and genetic information, can potentially overcome the limitations of current prognostic models and pave the way for more precise, personalized treatment strategies, ultimately leading to improved patient outcomes.

AI-based techniques, such as machine learning, have been increasingly applied to various fields of medicine, including cancer research, to enhance the accuracy of diagnosis, treatment selection, and prognosis prediction^13^. Utilizing multimodal data as input for AI-based algorithms could be a novel and groundbreaking approach for survival prediction. However, few methods have been proposed to fully exploit the potential of multiple data modalities^8^.

The primary objective of our study is to enhance the prognostic prediction in OSCC by leveraging multimodal data encompassing clinical, histological, and genetic information. To achieve this, we first undertook a thorough exploration of gene expression profiles and biological processes in OSCC. This initial phase involves comprehensive gene expression analyses, principal component analyses, gene enrichment studies and feature selection. These steps are pivotal in identifying key genetic features that might underpin OSCC pathogenesis, offering critical insights into the disease's complexity. Subsequently, we employ these insights to inform our machine learning and deep learning models. By first establishing a deep understanding of the underlying genetic and histopathological landscape, our approach aims to refine the selection of features that are most indicative of survival outcomes. This methodical progression from fundamental gene expression studies to the application of advanced AI techniques is designed to ensure that the resulting models are not only technically robust but also grounded in clinically relevant biological insights. The results of this study have the potential to provide novel insights into the development of prognostic and predictive biomarkers for OSCC, which can aid in the development of more personalized treatment plans and improve patient outcomes.

## Methods

### Study design

The original datasets comparing the gene expression profiles between solid, healthy, and solid tumor tissue were obtained from the National Cancer Institute GDC Data Portal (https://portal.gdc.cancer.gov/). All data that were processed were from the TCGA-HNSC project, which included only head and neck squamous cell carcinomas^14^. TCGA utilizes a strict set of criteria for inclusion into the study due to the rigorous and comprehensive nature of the work being performed. Tissue samples from tumors and their corresponding germline DNA sources are collected and handled by the Centralized Biorepository, a dedicated facility responsible for examining specimen information and processing all samples to maintain uniform pathology evaluation and production of molecular elements (DNA and RNA). Upon arrival at the Centralized Biorepository, every sample undergoes a stringent quality assurance process before being approved for comprehensive analysis within the TCGA workflow. A pathologist examines each specimen to verify the diagnosis and ensure it fulfills the inclusion criteria. Specifically, TCGA mandates that samples possess a minimum of 60% tumor nuclei and no more than 20% necrotic tissue. Once a sample clears the pathological assessment, nucleic acids are extracted, and genotyping is carried out to accurately link each tumor specimen with its corresponding normal tissue. An important goal in establishing this central resource is to ensure that molecular analytes (i.e., DNA and RNA) extracted from tissue samples are of consistent and high quality. Next, these analytes undergo a molecular quality control process and then are distributed to TCGA Cancer Genome Characterization Centers and Genome Sequencing Centers for genomic analysis. All samples in TCGA have been collected and utilized following strict policies and guidelines for the protection of human subjects, informed consent, and IRB review of protocols^14^. Inclusion criteria for the present study following the extraction of the initial TCGA-HNSC dataset were OSCC and patients who had histopathology and genetics data available. In alignment with previous prognostic research on OSCC utilizing the TCGA-HNSC dataset^15,16^, only the following sites were included: alveolar ridge, base of tongue, buccal mucosa, floor of mouth, hard palate, hypopharynx, lip, oral cavity, oral tongue, oropharynx, and tonsil. No additional exclusion criteria were applied beyond these parameters, allowing for a comprehensive and representative sample of OSCC patients for our analyses.

### Image processing

**Figure 1:**
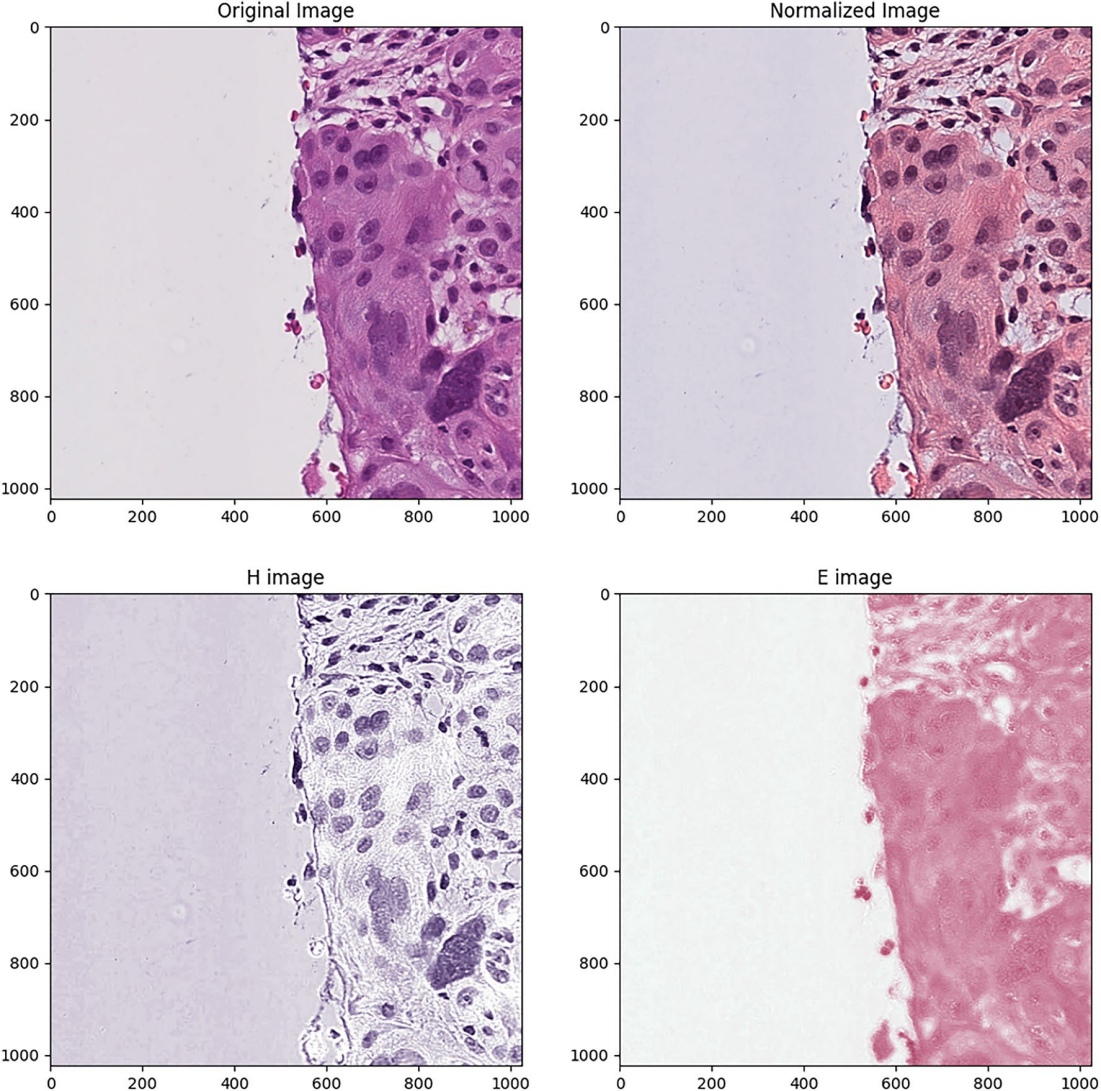
Representative examples of a non-normalized tile, a normalized tile, the Hematoxylin (H)-stained tile, and the Eosin (E)-stained tile. The non-normalized tile represents the original raw image tile extracted from the digital whole slide image. The H tile is generated by first converting the original histology tile to grayscale and then applying a high-pass filter, which enhances the high-frequency information in the image. This results in an image with a blue-purple hue, as hematoxylin stains the nuclei of cells in shades of blue. The H tile emphasizes the cell nuclei, which contain important diagnostic information. The E tile, on the other hand, is generated by first converting the original histology tile to grayscale and then applying a low-pass filter, which retains the low-frequency information in the image. This results in an image with a pink-orange hue, as eosin stains the cytoplasm and extracellular matrix in shades of pink. The E tile emphasizes the tissue structure and texture, which can provide additional diagnostic information. The normalized tile is the result of the normalization of the tile to reduce color variation between slides and was used for further analyses. The figure was generated using Python (version 3.10.4).

Digitized whole-slide images of H&E-stained specimens from primary untreated tumors were processed to extract quantitative histological features, sourced from the TCGA database. Employing a custom Python script and the OpenSlide library, we applied color normalization techniques to these images, following the methodologies described by Macenko et al. and implemented in Python by Vahadane et al.^17,18^. This process ensured consistent color representation across slides. To facilitate feature analysis, images were segmented into tiles of 1024 by 1024 pixels, focusing on areas with the highest density of diagnostic information as identified in previous research^19^. Figure 1 illustrates the normalization of a random tile by the algorithm. Using CellProfiler^20^, we extracted 170 quantitative features from these tiles, including metrics related to cell shape, size, texture, and pixel intensity distributions. This multi-dimensional data was then integrated with genomic and clinical information for comprehensive analysis. Detailed methodologies and scripts used for image processing are available in the Supplementary Material.

### Genomics analyses

Our genomic analysis utilized RNA-Seq data from the TCGA-HNSC project, focusing on primary tumor and normal tissue samples. Data preprocessing and analysis were conducted using the TCGAbiolinks package in R, employing a series of steps to ensure data quality and relevance. Lowly expressed genes were filtered out using the filterByExpr method in the limma package to concentrate on genes with significant expression levels. The TMM method followed by the voom transformation was applied for normalization, adjusting for library compositional differences and preparing data for linear modeling. Employing linear modeling and empirical Bayes statistics, we identified the top 200 differentially expressed genes. These genes were further analyzed through PCA to visualize variance and clustering, aiding in distinguishing between tumor and healthy tissue samples. To assess gene expression's impact on survival, we applied the Elastic Net algorithm^21^. Elastic Net, with its dual advantages of Lasso's feature selection and Ridge's multicollinearity management, provides a balanced approach that enhanced both the interpretability and robustness for predictions^22^, making it especially suited for the complex nature of OSCC gene expression data. The analysis led to the identification of 72 predictive genes, visualized through a heatmap created with the heatmap.2 function from the gplots package, highlighting the expression patterns between normal and tumor samples.

Gene enrichment analysis was conducted using the DAVID (Database for Annotation, Visualization, and Integrated Discovery) bioinformatics database to identify significant GO terms and KEGG pathways^23–25^ among the differentially expressed genes, setting a significance threshold at *p* < 0.05. This comprehensive genomic analysis approach, detailed further in the Supplementary Material, allowed for the robust identification and visualization of key genes and pathways relevant to OSCC.

### Statistical analyses and artificial intelligence-based techniques

Our analysis utilized a combination of statistical methods and artificial intelligence-based techniques, executed in R (version 3.2.3), Python (version 3.10.4), and SPSS Modeler. Supported by high-performance computing, including an AMD Ryzen 9 5950X processor and NVIDIA GeForce RTX 3090 GPU, we processed and analyzed OSCC data for predictive modeling and survival analysis. Data preprocessing, involving cleaning and normalization, was conducted using scikit-learn and Pandas libraries. We employed survival prediction models such as Random Survival Forest, Gradient Boosting Survival Analysis, Survival Support Vector Machine, Cox proportional hazards model, and a custom-developed deep learning model in Keras, focusing on the Cox model's negative log partial likelihood for patient outcome prediction. Model performance evaluation was based on the concordance index (C-index), with feature importance assessed through a c-index reduction approach to refine model predictions. We utilized a comprehensive strategy to address model overfitting and selection bias, incorporating regularization techniques, manual hyperparameter tuning, and k-fold cross-validation. This analytical framework facilitated the integration of clinical, histological, and genetic data into our models. For a detailed description of the data preprocessing steps, model development, and evaluation criteria, refer to the Supplementary Material.

## Results

### Descriptive statistics

**Figure 2:**
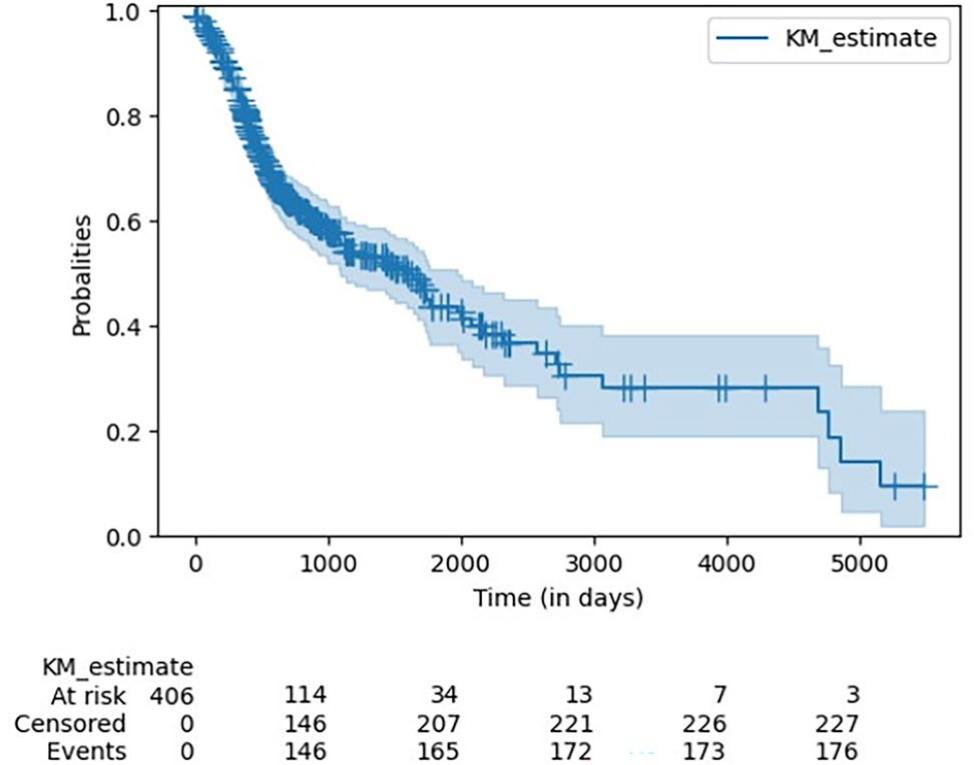
Kaplan–Meier survival curve of the cohort. The figure was generated using Python (version 3.10.4).

Table 1 illustrates the descriptive statistics of the analyzed TCGA dataset. A total of 406 OSCC patients were analyzed. N = 294 (72.41%) were male, and n = 112 (27.59%) were female. The mean age of patients at the time of diagnosis was 61.53 ± 12.38 years. The majority of patients were classified as "white race" (n = 354; 87.19%), followed by "black or African American" (n = 29; 7.14%). The most frequent pathological (n = 196; 48.28%) and clinical (n = 203; 50.00%) stage was IV A. N = 17 (4.19%) patients had prior malignancies, and n = 8 (1.97%) received prior treatment. N = 139 (34.24%) had no signs of pathological lymph node metastases, and n = 145 (35.71%) were classified as M0 based on pathological AJCC staging. Figure 2 shows the Kaplan–Meier survival curve and the risk table of the cohort. Time: in days. The median Survival estimate according to the Kaplan–Meier-Method was 1591 days (95% CI 1199.89–1982.11).

**Table 1:** Descriptive statistics of clinical features of patients (n = 406).

Variable	Count (%)	Mean ± SD
Demographics		
Age at diagnosis		61.53 ± 12.38
Gender–Female	112 (27.59%)	
Gender–Male	294 (72.41%)	
Race–Black or African American	29 (7.14%)	
Race–White	354 (87.19%)	
Race–Asian	10 (2.46%)	
Race–American Indian or Alaska Native	1 (0.25%)	
Race–Missing	12 (2.96%)	
Ethnicity–Not Hispanic or Latino	360 (88.67%)	
Ethnicity–Hispanic or Latino	19 (4.68%)	
Ethnicity–Missing	27 (6.65%)	
Lifestyle factors and previous malignancy		
Cigarettes per day		1.30 ± 1.91
Years smoked		8.74 ± 15.86
Pack years smoked		23.71 ± 34.87
Alcohol history—No	130 (32.02%)	
Alcohol history—Yes	266 (65.52%)	
Alcohol history—Missing	10 (2.46%)	
Prior malignancy—No	389 (95.81%)	
Prior malignancy—Yes	17 (4.19%)	
Clinical staging		
AJCC clinical stage—Stage I	15 (3.69%)	
AJCC clinical stage—Stage II	88 (21.67%)	
AJCC clinical stage—Stage III	80 (19.70%)	
AJCC clinical stage—	203 (50.00%)	
AJCC clinical stage–Stage IVB	5 (1.23%)	
AJCC clinical stage—Stage IVC	4 (0.99%)	
AJCC clinical stage–Missing	11 (2.71%)	
AJCC clinical T–T1	29 (7.14%)	
AJCC clinical T–T2	130 (32.02%)	
AJCC clinical T–T3	97 (23.89%)	
AJCC clinical T–T4	22 (5.42)	
AJCC clinical T–T4a	114 (28.08%)	
AJCC clinical T–T4b	2 (0.49%)	
AJCC clinical T–TX	9 (2.22%)	
AJCC clinical T–Missing	3 (0.74%)	
AJCC clinical N–N0	195 (48.03%)	
AJCC clinical N–N1	66 (16.26%)	
AJCC clinical N–N2	15 (3.69%)	
AJCC clinical N–N2a	13 (3.20%)	
AJCC clinical N–N2b	63 (15.52%)	
AJCC clinical N–N2c	31 (7.64%)	
AJCC clinical N–N3	4 (0.99%)	
AJCC clinical N–NX	16 (3.94%)	
AJCC clinical N–Missing	3 (0.74%)	
AJCC clinical M–M0	383 (94.33%)	
AJCC clinical M–M1	3 (0.74%)	
AJCC clinical M–MX	17 (4.19%)	
AJCC clinical M–Missing	3 (0.74%)	
Pathological staging		
AJCC pathologic stage–Stage I	23 (5.67%)	
AJCC pathologic stage–Stage II	61 (15.02%)	
AJCC pathologic stage–Stage III	70 (17.24%)	
AJCC pathologic stage–Stage IVA	196 (48.28%)	
AJCC pathologic stage–Stage IVB	8 (1.97%)	
AJCC pathologic stage–Missing	48 (11.82)	
AJCC pathologic T–T1	38 (9.36%)	
AJCC pathologic T–T2	120 (29.56%)	
AJCC pathologic T–T3	79 (19.46%)	
AJCC pathologic T–T4	11 (2.71%)	
AJCC pathologic T–T4a	114 (28.08%)	
AJCC pathologic T–T4b	4 (0.99%)	
AJCC pathologic T–TX	21 (5.17%)	
AJCC pathologic T–Missing	19 (4.68%)	
AJCC pathologic N–N0	139 (34.24%)	
AJCC pathologic N–N1	56 (13.79%)	
AJCC pathologic N–N2	11 (2.71%)	
AJCC pathologic N–N2a	4 (0.99%)	
AJCC pathologic N–N2b	85 (20.94%)	
AJCC pathologic N–N2c	34 (8.37%)	
AJCC pathologic N–N3	4 (0.99%)	
AJCC pathologic N–NX	52 (12.81%)	
AJCC pathologic N–Missing	21 (5.17%)	
AJCC pathologic M–M0	145 (35.71%)	
AJCC pathologic M–MX	52 (12.81%)	
AJCC pathologic M–Not reported	209 (51.48%)	
Prior treatment		
Prior treatment–No	398 (98.03%)	
Prior treatment–Yes	8 (1.97%)	
Outcome		
Vital status–Dead	177 (43.60%)	
Vital status–Alive	229 (56.40%)	
Overall survival		878.87 ± 857.18

### Comprehensive analysis of gene expression profiles: identifying key differentially expressed genes and pathways in OSCC

**Figure 3:**
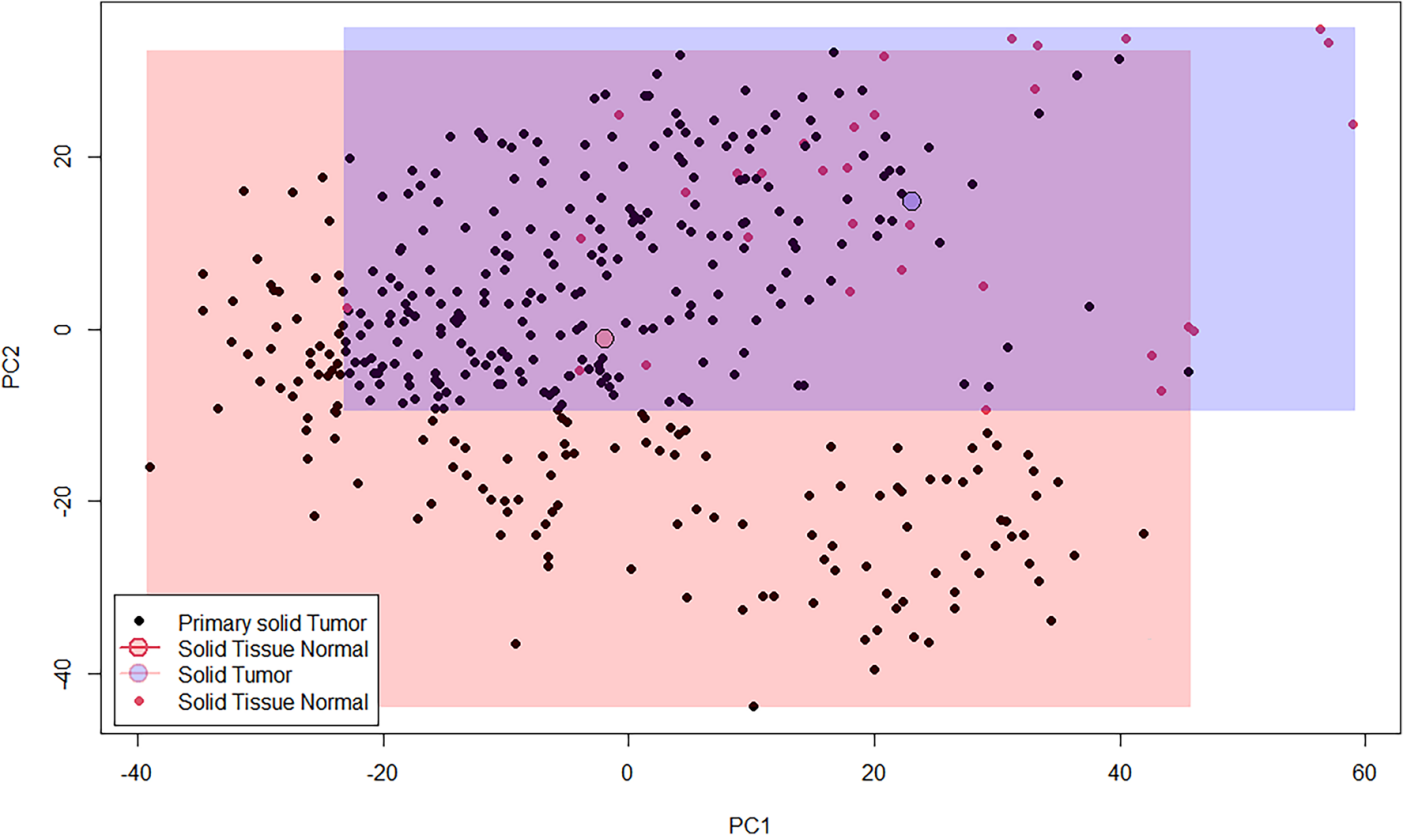
Principal component analysis (PCA) of differentially expressed genes. Each principal component (PC) represents a linear combination of the original variables (gene expression levels) and is orthogonal to the other components. PC1 and PC2 are the two linear combinations of the gene expression data that explain the most variation in the dataset. The axes in a PCA plot represent the principal components. The x-axis represents the first principal component (PC1) and the y-axis represents the second principal component (PC2). Each point in the plot corresponds to a sample, and its position along the axes represents its scores on the principal components. Points that are close together on the plot have similar gene expression profiles, while points that are further apart have more distinct profiles. The rectangles represent the boundaries of each group along the two principal components. The two average circles in the PCA analysis represent the centroids of the two groups. They show the average position of the data points in each group along the first two principal components. The figure was generated using Python (version 3.10.4).

**Figure 4:**
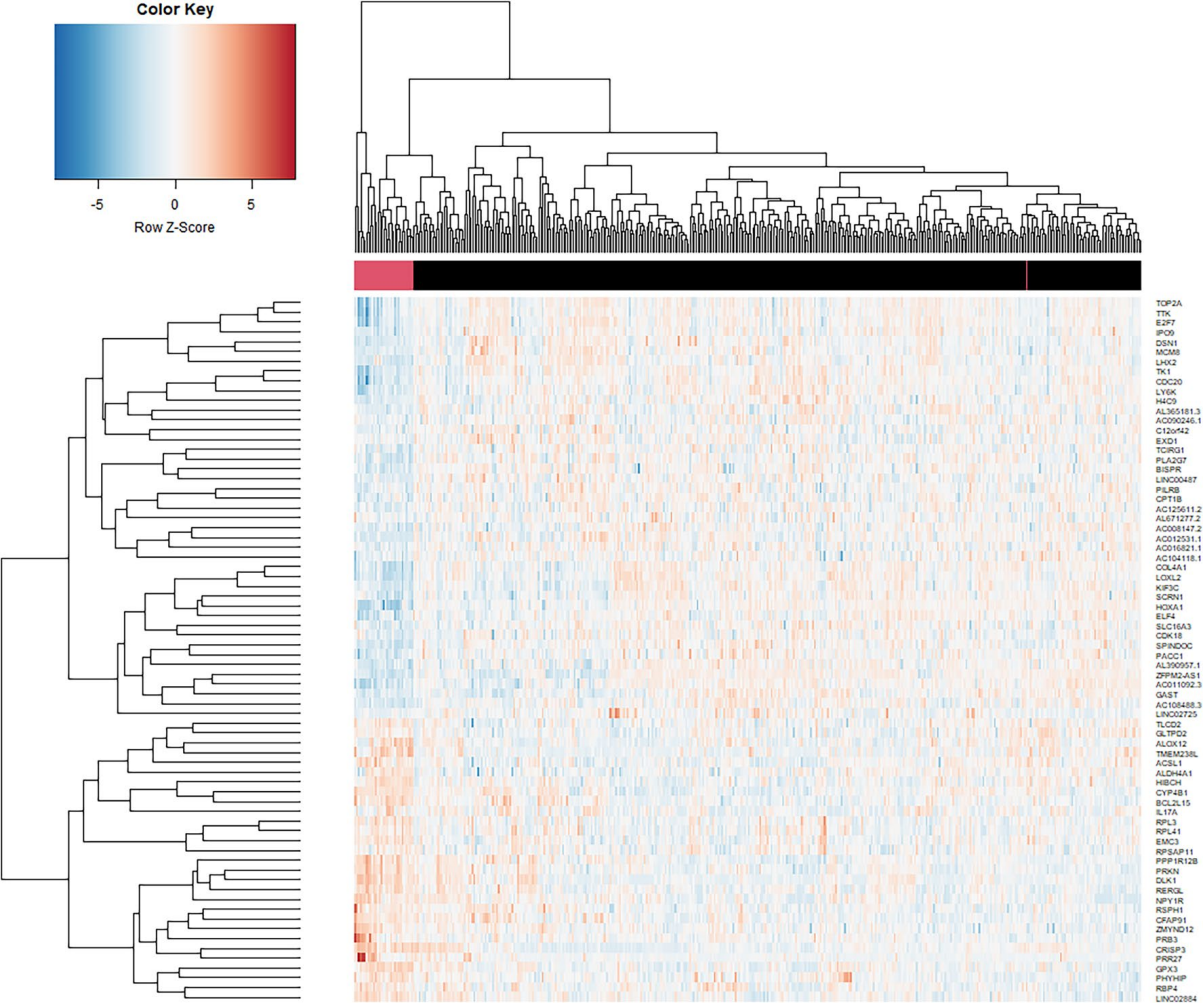
Heatmap of top differentially expressed genes (n = 72) as identified by ElasticNet. The columns represent the normal (red) and tumor solid tissue (black). The color scale ranges from blue (indicating low expression) to red (indicating high expression). The dendrogram for the rows (genes) of the heatmap represents the hierarchical clustering of genes based on their similarity in expression across the samples. The dendrogram for the columns (samples) of the heatmap represents the hierarchical clustering of samples based on their similarity in expression across the genes. The dendrograms show how similar or dissimilar the samples or genes are to each other based on their expression patterns. The height of the dendrogram represents the distance between clusters, with shorter distances indicating greater similarity or correlation. Clusters that are more similar are grouped together and have a common color in the heatmap. A total of 200 DEGs were analyzed further and are not shown here for better visualization. The figure was generated using Python (version 3.10.4).

Figure 3 highlights the results of the PCA. The two average circles in the PCA analysis represent the centroids of the two groups. They show the average position of the data points in each group along the first two principal components. The centroid is calculated by taking the mean of the x and y coordinates of all the data points in the group. As the circles are far apart, it suggests that the two groups are well separated along the first two principal components, which is a sign of differential gene expression between the two groups. Figure 4 highlights the top differentially expressed genes that were obtained through the comparison of solid normal and tumor tissue by the ElasticNet model. The further analyses contained a total of 200 differentially expressed genes that were assessed solely for the tumor tissue samples.

**Figure 5:**
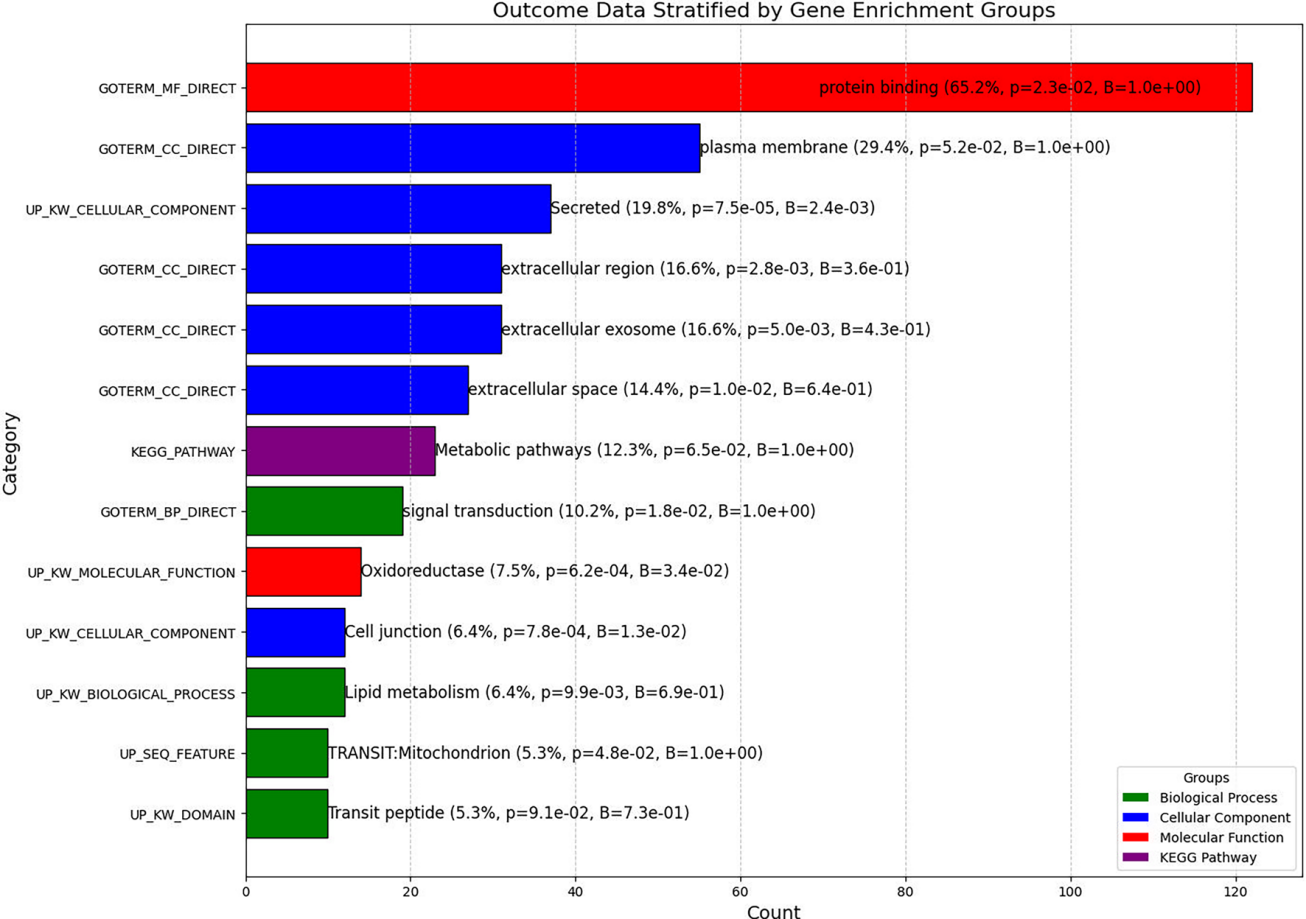
Results of the gene enrichment analyses. Genes with a total count of ≥ 10 are shown. Gene Ontology (GO) terms and Kyoto Encyclopedia of Genes and Genomes (KEGG) pathways were used as functional annotation categories. The enrichment analysis was performed with the default parameters, and the significance threshold was set at *p* < 0.05. The resulting output included enriched terms, count (%), their corresponding *p*-values, and Benjamini-corrected p-values. The figure was generated using Python (version 3.10.4) based on data from KEGG^23–25^.

The results of the gene enrichment analyses are shown in Fig. 5. The results showed that several biological processes and molecular functions were significantly enriched. The most enriched molecular function was protein binding, which was identified in 65.2% of the analyzed genes. The cellular component analysis revealed that the plasma membrane, secreted proteins, and extracellular regions were highly represented. Interestingly, metabolic pathways were also enriched, suggesting a possible link between metabolic processes and OSCC development that was also suggested recently through genomics analyses^26^. In addition, lipid metabolism, oxidoreductase activity, and cell junction were also found to be enriched.

### Evaluating prognostic factors and model performance in ai-based oscc survival prediction

**Figure 6:**
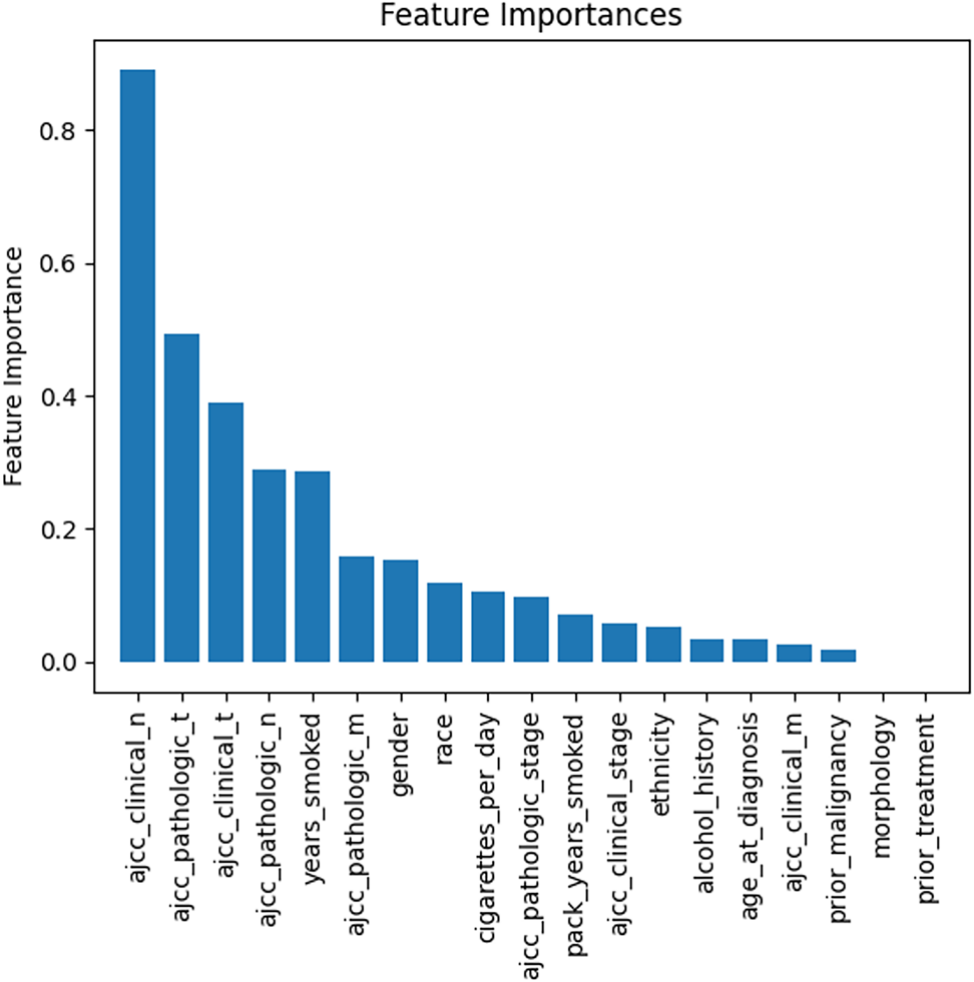
Feature importance analysis for the clinical variables. The figure was generated using Python (version 3.10.4).

The results of the feature importance analyses for clinical features are shown in Fig. 6. As expected, the AJCC staging variables were the most significant predictors of survival. Furthermore, smoking and gender were among the top 10 predictors. This confirms prior knowledge that smoking, and gender are important predictors of survival^27–29^.

Table 2 shows the comparison of unimodal and multimodal artificial intelligence-based analyses for survival prediction. We assessed the performance of unimodal and multimodal models in predicting patient outcomes using the c-index metric. The unimodal models included clinical, pathology, or genetic features, while the multimodal model combined all three types of features. The results showed that the multimodal model outperformed the unimodal models across all methods, with c-index values of 0.722 for RSF, 0.633 for GBSA, 0.625 for FastSVM, 0.633 for CoxPH, and 0.515 for DeepSurv. When considering only important features, the multimodal model continued to outperform the unimodal models, with c-index values of 0.834 for RSF, 0.747 for GBSA, 0.718 for FastSVM, 0.742 for CoxPH, and 0.635 for DeepSurv. The important features in the multimodal model were ENSG00000150667.8 (fibrous sheath interacting protein 1), ENSG00000186868.16 (microtubule associated protein tau), ENSG00000119147.10 (ECRG4 augurin precusor), ENSG00000272540.1 (novel transcript antisense to TUBB), ENSG00000105929.16 (ATPase H + transporting V0 subunit a4), ENSG00000124203.6 (zinc finger protein 831), ENSG00000172340.15 (succinate-CoA ligase GDP-forming subunit beta), Intensity MinIntensity Eosin (minimum pixel intensity values for Eosin staining), years of smoking, and AJCC clinical N-staging. These results suggest that combining clinical, pathology, and genetic features improves the accuracy of predicting patient outcomes compared to using each feature type alone.

**Table 2:** Unimodal and multimodal artificial intelligence-based analyses for survival prediction.

		Clinical	Pathology	Genetics	Multimodal
All features	RSF	0.714	0.530	0.529	0.722
	GBSA	0.691	0.539	0.542	0.633
	FastSVM	0.684	0.489	0.527	0.625
	CoxPH	0.686	0.503	0.547	0.633
	DeepSurv	0.462	0.538	0.501	0.515
Important features	RSF	0.698	0.635	0.637	0.834
	GBSA	0.672	0.568	0.593	0.747
	FastSVM	0.706	0.500	0.636	0.718
	CoxPH	0.708	0.510	0.632	0.742
	DeepSurv	0.413	0.557	0.503	0.635

The values represent the c-index. The c-index is a commonly used metric in survival analysis that evaluates the predictive accuracy of a model. It measures the probability that, given two randomly selected patients, the patient with the worse prognosis, according to the model, will experience an event (such as death) before the patient with the better prognosis. A c-index of 0.5 indicates that the model is no better than a random chance at predicting outcomes, while a c-index of 1.0 indicates perfect predictive accuracy.

**Figure 7:**
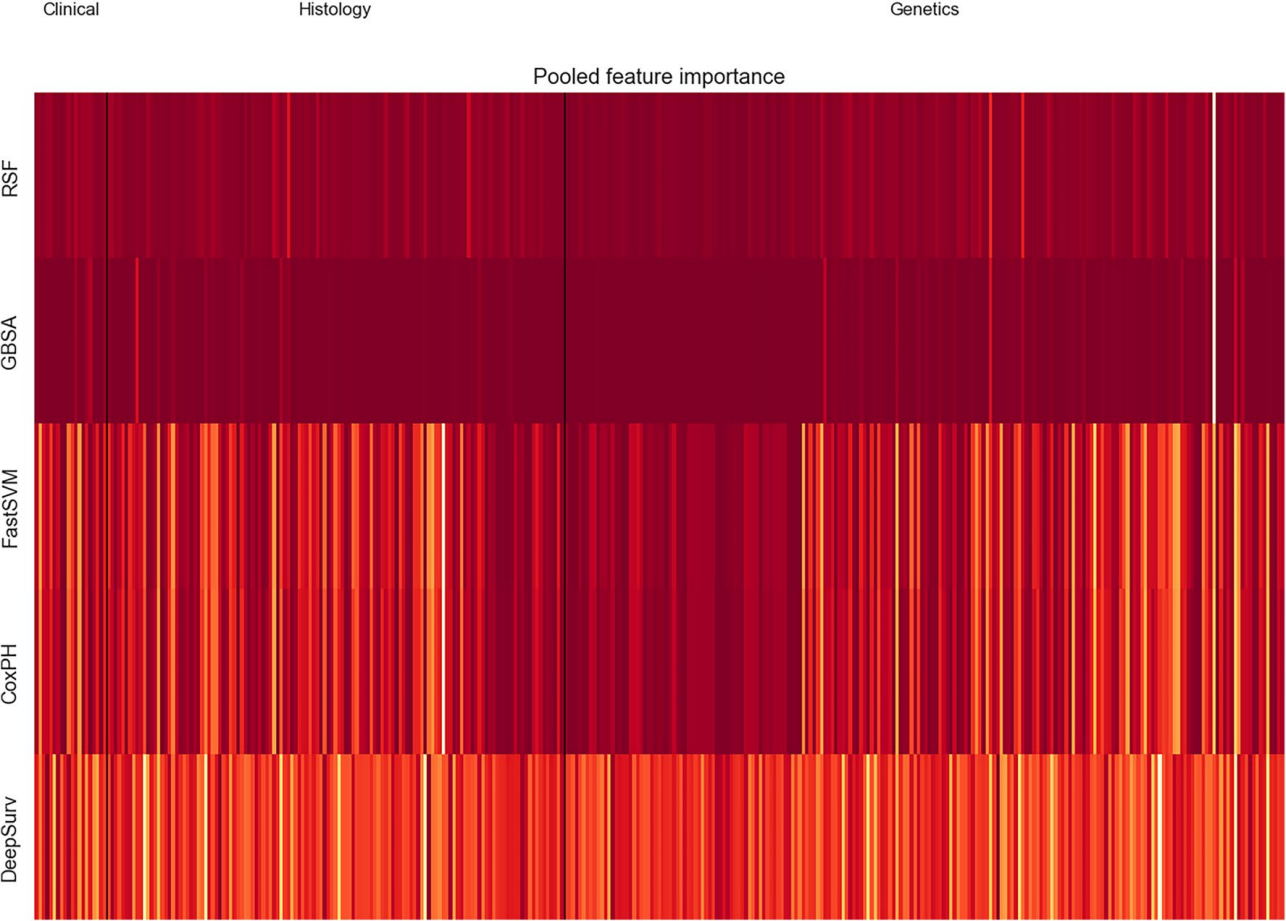
The heatmap displays the pooled feature importance scores for all models in our analysis. The rows represent different machine learning and deep learning models, and the columns represent the features (i.e., variables) used in each model. The features were further stratified into clinical, histological, and genetic features (separated by black vertical lines). The colors in the heatmap reflect the importance scores, ranging from dark red (highest importance) to yellow (lowest importance). The figure was generated using Python (version 3.10.4).

Figure 7 illustrates the pooled multimodal feature importance as evaluated by the models. The heatmap displays the pooled feature importance scores for all models in our analysis. The rows represent different machine learning models, and the columns represent the features (i.e., variables) used in each model. The features were further stratified into clinical, histological, and genetic features. The colors in the heatmap reflect the importance scores, ranging from dark red (highest importance) to yellow (lowest importance). The importance scores were calculated using permutation feature importance, which is a technique that evaluates the importance of each feature by randomly permuting its values and measuring the impact on the model's performance. The resulting importance scores were then scaled between 0 and 1 for each model so that the scores are comparable across models. We can see that some features have consistently high importance across all models, while others have variable importance depending on the model. This suggests that some features may be more robust and informative for predicting survival outcomes than others, justifying the evaluation of the c-index for both all features and important features solely in Table 2.

## Discussion

The present study included multimodal data (genomics, pathology, and clinical features) for survival prediction in OSCC patients. Our results provide evidence of improved prediction capacity by incorporating more patient information in prediction tasks for survival prediction in OSCC patients.

In this study, we employed a combination of sophisticated models, including the Cox Proportional Hazards (CPH) model implemented by CoxPHSurvivalAnalysis from sksurv.linear_model, as well as advanced machine learning models such as RandomSurvivalForest and GradientBoostingSurvivalAnalysis from sksurv.ensemble, FastSurvivalSVM from sksurv.svm, and KerasRegressor from keras.wrappers.scikit_learn. This approach aimed to leverage the strengths of traditional hazards-based models while also exploring the potential benefits of using more advanced machine learning and deep learning techniques for outcome prediction in cancer patients. While the traditional CPH model is useful for inferring the impact of variables on survival curves, integrating machine learning and deep learning methods can further enhance predictive accuracy. Artificial intelligence-driven approaches emphasize prediction over explanation and can address challenges like nonlinear gene interactions and multicollinearity, which may pose difficulties for conventional statistical methods. By examining extensive data, encompassing factors such as disease status, pathology, and genetic profiles, machine learning and deep learning models can determine the most advantageous treatment or clinical trial for a patient. Traditional statistical analyses may struggle with multicollinearity, particularly when integrating new prognostic factors. However, specific machine learning algorithms remain unaffected by significant collinearity among variables and can manage high-dimensional data^30^. For instance, Random Survival Forest (RSF) has outperformed classic CPH regressions in multiple studies^31–33^. Additionally, deep learning neural networks have demonstrated enhanced predictive accuracy compared to the traditional CPH model^34–36^. In a prior study, a nomogram predicting survival based on clinical variables and molecular markers for 68 oral SCC patients (validation dataset) achieved a c-index of 0.697, similar to the CoxPHSurvivalAnalysis result in this study^37^. Notably, RSF and deep learning models showed further improvements. The c-index serves as an excellent survival performance metric, as it is independent of a single fixed evaluation interval and considers censoring. The C-index's ability to handle censored data effectively is particularly pivotal in analyzing OSCC datasets, where such data is prevalent. Furthermore, its integration with our feature importance analysis, especially through the C-index reduction technique, enriches the interpretability and clinical applicability of our model. This approach, favoring the C-index over time-dependent AUC, aligns our work more closely with the practical demands and standards of clinical prognosis in OSCC. Our methodology showcases the potential to boost predictive accuracy in cancer patient outcomes beyond the capabilities of traditional statistical methods by employing a mix of advanced techniques.

Notably, there are several other techniques for multimodal data processing, and the present work applied only one of them (early fusion). In the field of multimodal fusion, prior research has investigated early and late fusion techniques. Early fusion concatenates features, while late fusion combines modalities through weighted averaging, failing to account for cross-modal interactions^38,39^. However, recent studies have demonstrated successful multimodal fusion through bilinear and graph-based models that exploit relationships within each modality^40,41^. Adversarial representation graph fusion (ARGF) has introduced a hierarchical interaction learning procedure, generating bimodal and trimodal interactions based on unimodal and bimodal dynamics^42^. Promising attempts have combined pathology and genomic data for cancer prognosis^43,44^. The Kronecker product, which creates a high-dimensional feature of quadratic expansion based on pairings of two input feature vectors, has demonstrated superior cancer survival prediction^40,45,46^. However, it may introduce a large number of parameters, increasing computational costs and risking overfitting^47,48^. Hierarchical factorized bilinear fusion for cancer survival prediction (HFBSurv) integrates genomic and image features, overcoming these limitations^49^. Recently, PONET was proposed at a scientific conference. PONET is an innovative biological pathway-driven pathology-genomic deep learning model that combines pathological images and genomic information to enhance survival prediction and pinpoint genes and pathways responsible for varying survival rates among patients^8^. Future validation of this model will provide information about its usefulness in clinics.

Despite the promising results obtained in this study, there are some limitations that need to be addressed. First, our study is based on retrospective data from the TCGA dataset, which may limit the generalizability of our findings to other cohorts or populations. In addition, the sample size of our study is relatively small, which may limit the statistical power and generalizability of our results. Further studies with larger sample sizes are needed to validate our findings. Moreover, the multimodal data processing approach used in our study requires sophisticated algorithms and computational resources, which may limit its feasibility for routine clinical practice. However, with the rapid advancements in computing power and AI technologies, the feasibility and practicality of this approach may improve in the future. Finally, our study is limited to the use of genomic, pathology, and clinical data, and other data modalities, such as radiomics and proteomics, were not included in the analysis. Future studies that incorporate multiple data modalities may provide a more comprehensive understanding of the disease and improve the accuracy of prognostic prediction.

## Conclusions

In this study, we present an approach for predicting the survival of OSCC cancer patients using multimodal data processing techniques. We have applied a stratification method to distinguish unimodal and multimodal data processing with regard to evaluation metrics. By using a multimodal data fusion technique, we evaluated several model architectures across multiple data modalities. Our results demonstrate that the use of multimodal data processing techniques can significantly improve the accuracy of predictive algorithms, leading to more accurate long-term survival predictions for patients with OSCC. These hybrid algorithms are capable of leveraging the rich and complex information provided by multiple high-dimensional data modalities in precision medicine-based clinical practices. By providing clinicians with accurate and reproducible predictions of patient prognosis, these algorithms hold great promise for enhancing the management of cancer patients.

### Supplementary Information

Supplementary Information.

## Data Availability

The codes and algorithm structures are available from: https://github.com/Freiburg-AI-Research. The raw data is publicly available from https://portal.gdc.cancer.gov/) (TCGA-HNSC dataset). Gene enrichtment analyses were conducted with data from Kyoto Encyclopedia of Genes and Genomes (KEGG)^23–25^.

## Abbreviations

OSCC: Oral squamous cell carcinoma
AI: Artificial intelligence
TCGA: The cancer genome atlas
H&E: Hematoxylin and eosin
HPV: Human papillomavirus
RSF: Random survival forest
GBSA: Gradient boosting survival analysis
Cox PH: Cox proportional hazards
FastSVM: Fast survival support vector machine
DeepSurv: Deep survival analysis
PCA: Principal component analysis
TCGAbiolinks: The cancer genome atlas bioinformatics links
GO: Gene ontology
KEGG: Kyoto encyclopedia of genes and genomes
DAVID: Database for annotation, visualization, and integrated discovery
SPSS: Statistical package for the social sciences
N: Number
CI: Confidence interval
AJCC: American joint committee on cancer
DE: Differentially expressed
GOTERM_MF_DIRECT: Gene ontology molecular function direct
GOTERM_CC_DIRECT: Gene ontology cellular component direct
UP_KW_CELLULAR_COMPONENT: UniProt keyword cellular component
KEGG_PATHWAY: Kyoto encyclopedia of genes and genomes pathway
GOTERM_BP_DIRECT: Gene ontology biological process direct
UP_KW_MOLECULAR_FUNCTION: UniProt keyword molecular function
UP_SEQ_FEATURE: UniProt sequence feature
UP_KW_DOMAIN: UniProt keyword domain
CPH: Cox proportional hazards
CoxPHSurvivalAnalysis: Implementation of cox proportional hazards model by CoxPHSurvivalAnalysis from sksurv.linear_model
sksurv: Scikit-survival
RandomSurvivalForest: Random survival forest model
GradientBoostingSurvivalAnalysis: Gradient boosting survival analysis model
FastSurvivalSVM: Fast survival support vector machine model
KerasRegressor: Keras regressor model
SCC: Squamous cell carcinoma
HFBSurv: Hierarchical factorized bilinear fusion for cancer survival prediction
