# Supplementary material for: Multimodal artificial intelligence-based pathogenomics improves survival prediction in oral squamous cell carcinoma
Source: Sci Rep. 2024 Mar 7;14:5687. doi: 10.1038/s41598-024-56172-5 (PMC10920832; doi:10.1038/s41598-024-56172-5)
Supplement: Supplementary file 1 — Supplementary Information. [file 41598_2024_56172_MOESM1_ESM.docx]

**Supplementary Methods**

**Image processing**

TCGA focuses on primary untreated tumors that were snap-frozen upon collection. Digitized whole-slide images of hematoxylin-and-eosin- (H&E-) stained specimens were obtained from TCGA. Processing of large histological images was carried out using a custom-made Python script and the OpenSlide library. The OpenSlide library was used to read large image files in the .svs format, and PIL (Python Imaging Library) was used to convert the image data to NumPy arrays. The NumPy arrays were then passed to a function, norm_HnE(), which implemented color normalization of H&E-stained histological images. The color normalization method was based on the approach described by Macenko et al. and implemented in Python by Vahadane et al. [1,2]. The norm_HnE() function first converted the RGB image to an optical density (OD) using the formula OD = −log10(I), where I is the image intensity. The OD values were then used to remove data with OD intensity less than β (0.15 in this case), estimate the covariance matrix of the remaining OD values, and compute the eigenvalues and eigenvectors using singular value decomposition (SVD). The two eigenvectors corresponding to the largest eigenvalues were then used to create a plane, onto which the OD values were projected and normalized to unit length. The angle of each point with respect to the first SVD direction was calculated, and the data were projected back to OD space to obtain the stain concentrations. The stain concentrations were then normalized to reference maximum stain concentrations for H&E, and extreme values were converted back to OD space to recreate the normalized image. The method separated the H and E components, allowing the analysis of each component separately. The DeepZoomGenerator class from the OpenSlide library was used to create a deep zoom image pyramid of the processed image, which was then saved as a .tif file using the tifffile library (version 2020.10.1).

Overall, we employed a quantitative approach to extract image features from pathological images available as large .svs files. This approach was chosen to normalize and divide images captured at 40x magnification into tiles of 1024 by 1024 pixels to reduce computational costs and maintain standardization. We then created a custom-made Python script to randomly select the top 10 tiles with the highest image density, defined as the summation of red, green, and blue values, based on previous research [3].

Using CellProfiler [4] and multiple modules within CellProfiler, such as "IdentifyPrimaryObject" and "IdentifySecondaryObject", we extracted a total of 170 quantitative features from the pathological images. These features encompassed several aspects, including shape, size, texture, and pixel intensity distribution of cells and nuclei. For each type of feature, the mean was calculated across all generated tiles and combined with the genomics and clinical data for further analysis using Python.

**Genomics analyses**

In the genomics analyses of our study, specific preprocessing techniques were meticulously applied to the raw gene expression data to ensure the accuracy and reliability of our results. The dataset was obtained using the TCGAbiolinks package in R, focusing on primary tumor and solid tissue normal samples from the TCGA-HNSC project. This dataset primarily involved RNA-Seq data with a workflow type of STAR-Counts. The preprocessing of this raw count data involved several key steps: 1)Gene Filtering: Initially, the data were filtered to remove lowly expressed genes, employing the filterByExpr method from the limma package. This step was crucial to focus on genes with significant expression levels, enhancing the robustness of subsequent analyses. 2)Normalization: Post-filtering, we normalized the data using the TMM (Trimmed Mean of M-values) method, followed by the voom method. The TMM normalization adjusted for compositional differences between libraries, while the voom method transformed RNA-Seq count data to log2-counts per million (logCPM), suitable for linear modeling. 3)Model Fitting and Statistical Analysis: After normalization, gene expression data were fitted using linear modeling techniques, followed by empirical Bayes statistics via the eBayes function. This approach allowed us to make inferences about differential expression, adjusting for the variability inherent in the data. 4)Differential Expression Analysis: As a final step, we identified the top 200 differentially expressed genes, which were then visualized using Principal Component Analysis (PCA). The PCA was conducted using the plot_PCA function, focusing on the first two principal components to illustrate the variance and clustering in the data. The colors and symbols in the plot represented the tumor and healthy solid tissue.

To evaluate the effect of gene expression on survival as an initial analysis step, we performed classification analysis using the Elastic Net algorithm, a regularized regression method. This choice was informed by the model's proficiency in handling high-dimensional data, typical in genomic studies, and its ability to address multicollinearity among predictors, a common challenge in gene expression profiling [5]. Furthermore, the Elastic Net, with its dual advantages of Lasso's feature selection and Ridge's multicollinearity management, provides a balanced approach that enhanced both the interpretability and robustness for predictions [6], making it especially suited for the complex nature of OSCC gene expression data. The elastic net was fit to the expression data using the 'glmnet' package in R, with a binomial response and an alpha parameter of 0.5. The dataset was randomly divided into a training set (75% of the data) and a test set (25% of the data). The model was trained on the training set using 10-fold cross-validation to determine the optimal value of lambda. The coefficients of the resulting model were then used to select genes that were most important for prediction. We considered only genes with non-zero coefficients as relevant for prediction. In our case, this resulted in 72 genes. These genes were then used to produce a heatmap to visualize their expression across the samples. The hierarchical clustering was performed using the heatmap.2() function from the gplots package to create a heatmap of the selected predictive genes (n=72) from the differentially expressed genes (n=200) between normal and primary tumor samples in the initial dataset. The color palette of the heatmap was defined, and complete linkage clustering was used to cluster the genes. The inverse of correlation was used as the distance metric, and the row scaling was performed. The heatmap included dendrograms for both axes, and the relevant predictive genes were labeled with their names instead of Ensembl annotation. The dendrograms on the top and left side of the heatmap were used to group the samples and genes, respectively, based on their expression patterns. The color scale ranged from blue (indicating low expression) to red (indicating high expression). Subsequent analyses included solely tumor samples.

DAVID (Database for Annotation, Visualization, and Integrated Discovery) bioinformatics database was used to perform gene enrichment analysis. The input for DAVID analysis was the list of differentially expressed genes obtained from the preprocessing step. Gene Ontology (GO) terms and Kyoto Encyclopedia of Genes and Genomes (KEGG) pathways [7–9] were used as functional annotation categories. The enrichment analysis was performed with the default parameters, and the significance threshold was set at p < 0.05. The resulting output included enriched terms, their corresponding p-values, and Benjamini-corrected p-values.

**Statistical analyses and artificial intelligence-based techniques**

All analyses were performed in R programming language (version 3.2.3), Python (version 3.10.4), SPSS modeler (v18.3, IBM Corp., Armonk, USA), and Python for Apache Spark framework within SPSS modeler, SPSS (v27, IBM Corp., Armonk, USA). In the development of our models, we utilized a combination of high-performance hardware and specialized software tools. Our computational infrastructure was anchored by an AMD Ryzen 9 5950X 16-Core Processor, complemented by 64 GB of RAM and an NVIDIA Geforce RTX 3090 GPU. This setup provided the necessary computational power for intensive data processing and model training tasks. On the software front, our analyses were primarily conducted using Python (version 3.10.4) and R, chosen for their extensive range of libraries and tools adept in handling complex data analysis and machine learning tasks. In Python, we employed libraries such as pandas, numpy, matplotlib, seaborn, lifelines, scikit-learn, tensorflow, and keras for data manipulation, visualization, survival analysis, and deep learning model development. In R, we leveraged TCGAbiolinks for TCGA data processing, ggplot for visualization, and packages like limma, edgeR, caret, and survminer for gene expression data analysis and statistical modeling. RNA-seq libraries were sequenced using the Illumina HiSeq 2,000 platform (Indexed, 76bp, paired-end run). OSCC genetics data were obtained via TCGAbiolinks, an R/Bioconductor package for integrative analysis with GDC data. Gene expression normalization and differential expression gene analyses were performed using the limma package and edgeR package in R (Ritchie et al., 2015). Candidate genes from tumor patients were then concatenated into the multimodal dataset for further processing using Python.

First, the data were preprocessed using various functions from the scikit-learn and Pandas libraries. The missing values in the categorical variables were imputed using the "SimpleImputer" function with the strategy set to "most frequent". Specifically, for the AJCC Pathological M category, cases with non-assigned values were categorized under a distinct group labeled "Not Reported", to acknowledge the clinical scenario where pathological metastasis status might not be reported due to the absence of clinical indications for distant metastasis. This categorization was made to ensure the integrity of our analysis by distinctly identifying and including cases with non-reported pathological M status. The categorical string variables were converted to numeric labels using the "LabelEncoder" function. The integer columns were converted to float64. The continuous variables were standardized using the "StandardScaler" function. The survival prediction models used in this study include Random Survival Forest, Gradient Boosting Survival Analysis, Survival Support Vector Machine, Cox proportional hazards model, and a deep learning model (“DeepSurv”). The deep learning model was implemented using Keras, a high-level neural networks API, with a TensorFlow backend. We defined a custom loss function, coxph_loss, that calculates the negative log partial likelihood of the Cox proportional hazards model. The deep survival model architecture consisted of a fully connected neural network with one hidden layer of 32 units and a ReLU activation function. The input dimension was set according to the number of features in the dataset. The output layer had two nodes with a softmax activation function to predict the probability of an event or censoring. The model was trained using the Adam optimizer and the coxph_loss function for 100 epochs. We used a batch size of 32 and a learning rate of 0.001.

The performance of the models was evaluated using the concordance index (C-index). To implement these models, the scikit-learn, sksurv, Keras, and lifelines libraries were used. The models were implemented using the following functions:

- RandomSurvivalForest from sksurv.ensemble
- GradientBoostingSurvivalAnalysis from sksurv.ensemble
- CoxPHSurvivalAnalysis from sksurv.linear_model
- FastSurvivalSVM from sksurv.svm
- KerasRegressor from keras.wrappers.scikit_learn

K-fold cross-validation (k=5) was used to evaluate the performance of the models. The evaluation metric used was the concordance index (c-index), which measures the ability of a model to correctly rank the survival times of patients. The c-index was calculated using the "concordance_index_censored" function from the sksurv.metrics library.

We performed feature importance analysis using the c-index reduction approach to identify the most important features for predicting the outcome of our study. The c-index measures the ability of a model to distinguish between pairs of subjects, one with an event and one without. A model with a c-index of 0.5 has no predictive power, while a model with a c-index of 1.0 has perfect predictive power. We first fitted baseline models using all available features and then sequentially removed each feature and measured the change in the c-index. We repeated this process until all features were removed from the model. The reduction in the c-index for each feature was then used to rank the importance of the features. We also used a bootstrap resampling approach to estimate the variability in the c-index reduction for each feature. The top features identified by the c-index reduction approach were then used to fit the final models. We assessed the performance of these models using the c-index. Overall, our feature importance analysis using the c-index reduction approach allowed us to identify the most important features for predicting the outcome of our study and to develop a more parsimonious and robust predictive model.

Overall, several robust methodologies were implemented to minimize the risks of model overfitting and selection bias. In addition to the feature importance analyses, we employed k-fold cross-validation (k=5) to enhance the generalizability of our models, ensuring each model's validation on unseen data. Regularization techniques used in the developing pipeline like Elastic Net helped prevent overfitting by penalizing model complexity. Model performance was rigorously evaluated using the concordance index (C-index) and Area Under the Curve (AUC) metrics at specific time points to ensure predictive accuracy over time. Furthermore, our models integrated a diverse set of data types—clinical, histological, and genetic—reducing the likelihood of selection bias and capturing OSCC's heterogeneity. Manual hyperparameter tuning, though time-consuming, was crucial in our study due to the relatively low number of hyperparameters. This meticulous manual process involved adjusting and evaluating different hyperparameter values to deeply understand their impact on model performance. It ensured optimal hyperparameter settings, further mitigating overfitting risks. Complementing this, we employed k-fold cross-validation techniques during hyperparameter selection to assess model performance across multiple data subsets. This comprehensive approach, blending manual tuning with cross-validation, was tailored to each model's specific characteristics, ensuring robustness and consistency in performance across different data splits. Standardization and appropriate preprocessing of the variables, including imputation strategies for missing data, maintained dataset quality and unbiased model training.

**References**

1. Macenko M, Niethammer M, Marron JS, Borland D, Woosley JT, Xiaojun Guan, et al. A method for normalizing histology slides for quantitative analysis. 2009 IEEE International Symposium on Biomedical Imaging: From Nano to Macro [Internet]. Boston, MA, USA: IEEE; 2009 [cited 2023 Apr 4]. p. 1107–10. Available from: http://ieeexplore.ieee.org/document/5193250/

2. Vahadane A, Peng T, Sethi A, Albarqouni S, Wang L, Baust M, et al. Structure-Preserving Color Normalization and Sparse Stain Separation for Histological Images. IEEE Trans Med Imaging. 2016;35:1962–71.

3. Salvi M, Acharya UR, Molinari F, Meiburger KM. The impact of pre- and post-image processing techniques on deep learning frameworks: A comprehensive review for digital pathology image analysis. Computers in Biology and Medicine. 2021;128:104129.

4. Carpenter AE, Jones TR, Lamprecht MR, Clarke C, Kang IH, Friman O, et al. CellProfiler: image analysis software for identifying and quantifying cell phenotypes. Genome Biology. 2006;7:R100.

5. Hughey JJ, Butte AJ. Robust meta-analysis of gene expression using the elastic net. Nucleic Acids Res. 2015;43:e79.

6. Tschodu D, Lippoldt J, Gottheil P, Wegscheider A-S, Käs JA, Niendorf A. Re-evaluation of publicly available gene-expression databases using machine-learning yields a maximum prognostic power in breast cancer. Sci Rep. 2023;13:16402.

7. Kanehisa M, Goto S. KEGG: kyoto encyclopedia of genes and genomes. Nucleic Acids Res. 2000;28:27–30.

8. Kanehisa M. Toward understanding the origin and evolution of cellular organisms. Protein Sci. 2019;28:1947–51.

9. Kanehisa M, Furumichi M, Sato Y, Kawashima M, Ishiguro-Watanabe M. KEGG for taxonomy-based analysis of pathways and genomes. Nucleic Acids Res. 2023;51:D587–92.
